# New York Letter

**Published:** 1896-09

**Authors:** L. B. Grandy

**Affiliations:** New York


### CORRESPONDENCE.

---

### MEDICAL NEW YORK.

NEW YORK, August 15, 1896.

The Reformer is abroad in Gotham, and much in evidence. The old-time Tammany Society, which for so long controlled this great city from center to circumference, has been retired, for the present at least, and the places that once knew it and flourished by it now know it no more. The Reform Government, whose *in hoc signo vincemus* was "Anything to Beat Tammany," is now on top, and in their wild desire to establish municipal cleanliness, rectitude, and honesty, they "stand so straight that they lean backwards." It is particularly questionable whether the efforts of Dr. Parkhurst to close up the so-called dens of prostitution have accomplished any desirable results. Few, I think, believe that this business can be stopped by any laws at present known to us. All, I am sure, know that it goes merrily on in New York, as before.

The spirit of reform is to be noted in New York's great Department of Charities as well as elsewhere. This department in times past, as well as the present, has been the *casus belli* for many dissensions, more or less spirited, among the local medical fraternity. The faculty of Bellevue Medical College once enjoyed the entire control and patronage of Bellevue Hospital, or the City Hospital. The University Medical College and the College of Physicians and Surgeons in time made a vigorous kick in behalf of their faculties and their demands were satisfied. Now another lively fight is on. A number of physicians in the city, not connected with the teaching faculty of either college, have united to demand what they conceive to be their rights in relation with the great quantity of clinical material in the city hospitals. The outcome of the contention is important to doctors here, and is being watched with interest.

The use of cocaine in surgical practice is becoming more and more general in New York, and the opinion prevails, at least among those who have given cocaine an extended trial, that many operations which are usually performed under general anesthetics may now be done easily and painlessly under the local. The use of Schleich's formulæ and of his method of injecting the cocaine solution has no doubt had much to do with this wider employment of local anesthesia, as it is his method, or some slight modification of it, that is being followed, as a rule. Dr. Wyeth, of the Polyclinic, and his assistant, Dr. Bodine, are especial advocates of cocaine anesthesia. Dr. Wyeth has operated for appendicitis and also for hernia under cocaine, and only lately he removed the entire larynx; besides tumors of various sizes and in various regions of the body; and all painlessly. This is somewhat introductory to the mention of an interesting case which I saw last week. A patient in the Polyclinic was thought to have a malignant growth of the larynx, which had encroached upon his rima glottidis and seriously reduced his breathing space. Tracheotomy was decided upon preparatory to a complete removal of the larynx. This was done by Dr. Bodine. Schleich's *medium* solution of cocaine was employed. (These formulæ were published in this JOURNAL about three or four months ago. The medium solution consists of one grain and a half of cocaine, one-third of a grain of morphine, three grains of sodium chloride, and three ounces of distilled water. Two or three drops of carbolic acid or five grains of boracic acid may be added to prevent decomposition.) This solution was freely injected *in* the skin, not *under* it, along the line of the proposed incision. The skin and underlying connecting tissue were divided and the muscles lying on either side of the median line were exposed and drawn aside. As soon as the trachea was bared a moment was allowed for all oozing to stop. Then it was the work of a second to divide the cricoid cartilage, and the next ring of the trachea. The whole was done absolutely without pain, and it made a very pretty and instructive illustration of what may be accomplished under cocaine anesthesia. It was intended to remove the larynx at yesterday's clinic, one week after the tracheotomy, but the relief of congestion which followed this operation had produced such a diminution in the size of the growth that it was decided to await further developments. It is impossible for a patient to absorb a poisonous quantity of cocaine when it is used according to the method of Schleich. It was estimated that the above patient did not receive more than a fiftieth of a grain, and yet this was sufficient, with the other constituents of the solution, to produce a complete anesthesia.

Speaking of cocaine one is reminded of the new local anesthetic, eucaine, which is attracting some attention here,particularly in ophthalmic practice. If all is true that is claimed for it, it will possess some distinct advantages over cocaine in ophthalmic work. The hydrochlorate is the salt most used, and is sparingly soluble in water. A few drops of a four per cent. solution in the eye is said to cause complete anesthesia in one to three minutes. It does not dilate the pupil. Eucaine is practically non-poisonous, and solutions of it do not undergo decomposition. It is to be hoped that further trials of eucaine will confirm the somewhat meager reports thus far made. The reader will perhaps be pleased to know that chemically eucaine is a methyl-benzoyl-tetramethyl-gamma-oxypiperidine-carbonic acid methyl-ester. At least I was told so.

Another new remedy which promises to be useful, especially in ophthalmic practice, is formalin. Heretofore this preparation has been employed principally as a germicide and preservative agent. Solutions of it, 1–100, are now being employed to touch corneal ulcers, and 1–2000 as a wash for purulent ophthalmia and trachoma. Solutions (1–3000) may be freely employed in the eye every hour. It does not precipitate albumen, nor does it injure instruments. The testimony of those who are using it is highly favorable to it as a valuable antiseptic in ophthalmic work. It is said to be much superior to the usual solutions of bichloride of mercury.

The *Medical Record* (Aug. 8th) contains a good article by Dr. R. Frothingham, of this city, upon the "Importance of an Understanding of Middle Ear Disease by All Practitioners," in which he calls attention to the fact that medical schools have as yet paid little attention to diseases of the ear. Consequently practitioners have gone forth to heal with little or no knowledge of this portion of their art. The author cites a number of illustrative cases first examined or treated by general practitioners who seemed to possess a woeful lack of knowledge of the primary principles of ear diseases.

It is to be hoped that in the boasted advances which we have been making in late years in medical teaching these mistakes will now be fewer and further between. The author makes a strong plea for a better understanding of the general principles of ear diseases on the part of practitioners.

Perhaps no operation is now established upon a more secure surgical and scientific basis than that of opening the mastoid cells for purulent middle ear disease. Where the operation can be properly carried out the results are usually all that can be hoped for in these serious conditions. I have had the pleasure of witnessing several of these operations in different institutions here, and a few days ago assisted in opening both mastoids in the same patient for a double acute purulent otitis of two weeks' duration. I am told that the result has been quite satisfactory.

Nowhere, so far as I know, have the indications for opening the mastoid been more clearly and succinctly set forth than in a late paper by Dr. Felix Cohn, read before the Manhattan Medical and Surgical Society. For the complete paper the reader is referred to the *New York Medical Journal*, August 8th. These indications, as the author conceives them, are stated briefly as follows: (1) all cases of diagnosticated osteitis which are not relieved by usual antiphlogistic measures; (2) cases of antral empyema which show no tendency to discharge completely through the middle ear; cases of protracted otitis with profuse otorrhea which shows no tendency to resolve within a reasonable period; the time chosen for operation depending upon the manifest symptoms, whether, for instance, retention is present or the mastoid bone itself is involved; (4) cases of acute otitis which present dangerous symptoms of resorption and in which proper drainage cannot be established by paracentesis or by the natural perforation; and this may be done for drainage and cleansing purposes even when no manifest symptoms of mastoid affection exist; (5) cases of muco-purulent otitis depending upon mastoid involvement; (6) cases of mastoid disease or otitis complicated by lymphangitis or lymphadenitis, in which there is imminent danger of the formation of an abscess, or in which the lymphadenitis does not tend to resolve under ordinary treatment; (7) cases of protracted otitis in which there are symptoms of serious secondary complications involving danger of extension of the inflammation inward toward the neck; (8) cases of acute otitis in which complicating stenosis of the external canal promotes drainage and thorough cleansing of the middle ear.

L. B. GRANDY, M.D.

---

### WHEN MAY GONORRHEAL PATIENTS MARRY?

Following are the conclusions of a paper on this subject by Dr. F. C. Valentine (*Am. Medico-Surgical Bulletin*):

1. Gonorrhea, *per se*, is a dangerous disease, but curable at any stage.

2. Even when all external evidences of its existence have disappeared, the patient may still be able to infect.

3. A woman infected with gonorrhea is in danger of her life.

4. No man should marry who can infect his wife.

5. In a week or ten days' time it can be determined whether a man can infect or not. This can be done by throwing some irritant injection into the urethra and examining the discharge for gonococci. If gonococci be present *the patient must not marry* until they are made to disappear absolutely and entirely.

---

### CONCENTRATED MILK.

A writer in the *British Medical Journal* has called attention to the value of concentrated milk in certain forms of diarrhea and in wasting disease, and especially in cases in which the patient is unable to take other nourishment, and cannot take a sufficient amount of milk in its ordinary diluted form to meet the demands of the body. Concentrated milk is prepared by evaporating the milk in a porcelain dish over some suitable heating apparatus, care being taken to see that the liquid does not boil and to stir it continually. By this means cream is prevented from rising, and the evaporation is not delayed by the formation of a scum over the surface. With proper apparatus and attention, milk may be reduced to one-half its volume in one hour.—*New York Medical Times*.

